# From verbal fluency to naturalistic speech: a scoping review of language correlates of loneliness and social isolation in older adults

**DOI:** 10.3389/fnagi.2026.1831374

**Published:** 2026-07-31

**Authors:** Maria Veiga de Araújo, Diana R. Pereira, Ana Paula Soares

**Affiliations:** Human Cognition Lab, CIPsi, School of Psychology, University of Minho, Braga, Portugal

**Keywords:** aging, cognition, language, linguistic processing, loneliness, social isolation, speech

## Abstract

**Introduction:**

Population aging poses significant societal and health challenges, with loneliness and social isolation (L/SI) recognized as pressing public health concerns. While social isolation refers to an objective lack of social contacts, loneliness reflects the subjective perception that one’s social needs are not being met. Although L/SI have been associated with an increased risk of dementia and global cognitive decline, their relationship with language functioning remains largely underexplored. Given the central role of language in social interaction, examining this relationship is particularly relevant.

**Methods:**

Following PRISMA-ScR guidelines, we conducted a scoping review to a) map the language domains and measures used in the literature, and b) synthesize evidence on the association between L/SI and language functioning in older adults. Web of Science, PubMed, and Scopus were searched without temporal restrictions using terms related to language, L/SI, and aging. Eligible studies included older adults (≥60 years) and examined the association between at least one language measure and one indicator of L/SI.

**Results:**

Thirty-four studies met the inclusion criteria. Considerable heterogeneity was observed in both language and social-disconnection measures. The literature focused primarily on verbal fluency tasks, with comparatively less attention devoted to lexical retrieval, discourse production, semantic processing, and speech-derived computational markers. Associations between L/SI and verbal fluency were suggestive but not robust after covariate adjustment, whereas discourse- and speech-derived markers emerged as promising, albeit methodologically underdeveloped, sources of evidence.

**Discussion:**

Current evidence suggests L/SI may be associated with language functioning in older adults. However, the literature remains conceptually and methodologically heterogeneous, highlighting the need for more systematic, comprehensive, and methodologically consistent research in this area.

## 1 Introduction

Demographic aging poses significant social, economic, and health challenges, with loneliness and social isolation (L/SI) recognized as an imperative public health issue (Cacioppo and Cacioppo, 2018a, 2018b; World Health Organization [WHO], 2021). Social isolation (SI) is defined as a structural and quantifiable lack of social contacts and relationships, whereas loneliness (L) is an individual’s subjective perception that their social needs are not being met, either in terms of the quantity or quality of their interactions (Cacioppo et al., 2011; Cacioppo and Cacioppo, 2018b; Heinrich and Gullone, 2006; Holt-Lunstad et al., 2015; Perissinotto et al., 2019). Although loneliness and social isolation are moderately correlated, they represent distinct constructs and do not necessarily co-occur (see Perissinotto et al., 2019 for a discussion). Individuals can feel lonely without being isolated, and conversely, they may not feel lonely even when they are socially isolated (Cacioppo et al., 2011; Holt-Lunstad et al., 2015). Although these experiences are not exclusive to older adults, they appear to be particularly vulnerable to L/SI for several factors, including limited socioeconomic resources, retirement, loss of close relationships, life-altering events, and health issues (Roy et al., 2023; WHO, 2021). L/SI have a negative impact on health, wellbeing, quality of life, and are even associated with mortality (Cacioppo and Cacioppo, 2018b; Holt-Lunstad et al., 2015; Roy et al., 2023; WHO, 2021). The impact on cognition is no exception.

More specifically, loneliness has been consistently associated with accelerated cognitive decline and has been identified as both a risk factor and predictor of dementia (Holwerda et al., 2014; Lara et al., 2019b; Wilson et al., 2007; Yin et al., 2019). It has been associated with poorer performance in global cognitive functioning and across several specific domains, including episodic memory, executive functions, processing speed, numerical reasoning, and semantic verbal fluency (Barnett et al., 2023; Boss et al., 2015; Harrington et al., 2023; Holwerda et al., 2012; Lee et al., 2025; Simpson et al., 2019; Wilson et al., 2007). Converging neuroimaging evidence further suggests that loneliness may be associated with structural brain alterations, including volumetric reductions in frontal white matter and in striatal regions such as the putamen and globus pallidus (Lee et al., 2024). Importantly, however, the effects of loneliness do not appear to be uniform across different stages of cognitive impairment. Moretti et al. (2024) showed that the association between loneliness and cognitive and functional status differed between individuals with major and minor neurocognitive disorders. After adjustment for relevant covariates, higher loneliness in patients with dementia were associated with poorer cognitive performance and reduced functional autonomy in both basic and instrumental activities of daily living. By contrast, among individuals with mild cognitive impairment, loneliness was positively associated with cognitive performance and functional autonomy. The authors suggested that, at earlier stages of cognitive decline, individuals may reveal more awareness of both loneliness and emerging cognitive difficulties, which could motivate compensatory efforts to preserve independence and seek cognitively stimulating activities.

Moreover, loneliness has frequently been conceptualized as a chronic psychosocial stressor and a risk factor for cognitive decline (Boss et al., 2015; Camacho et al., 2025; Lara et al., 2019b; Pollak et al., 2023). Prolonged loneliness has been associated with physiological stress responses, including dysregulation of the hypothalamic–pituitary–adrenal axis, increased inflammation, and alterations in brain structure and function, all of which may compromise neural integrity and increase vulnerability for neurodegenerative diseases (Cacioppo et al., 2011; White et al., 2015; Xia and Li, 2018). In addition to these direct stress-related mechanisms, loneliness may also affect cognition indirectly through physical health and health-related behaviors. For example, loneliness has been linked to less healthy patterns, such as reduced physical activity and poorer dietary habits, which may increase the risk of cardiovascular and metabolic conditions that are themselves associated with cognitive decline (Steptoe et al., 2004).

Social isolation has also been associated with poorer performance in overall cognition and in specific domains, including attention, executive functioning, perceptual speed, and visuospatial abilities (DiNapoli et al., 2014; Lara et al., 2019a; Rodriguez et al., 2017; Shankar et al., 2013). Additionally, living alone, having a limited social network, and reduced participation in social activities have been identified as predictors of faster decline in memory, verbal fluency, and global cognitive functioning (DiNapoli et al., 2014; Evans et al., 2019; Feng et al., 2025; Lara et al., 2019a; Penninkilampi et al., 2018; Rodriguez et al., 2017; Samtani et al., 2022; Shankar et al., 2013; Yin et al., 2019).

Social connection, which encompasses the structure, function, and quality of social relationships, plays a critical role in cognitive functioning in later life (Holt-Lunstad, 2022). Although both L/SI have adverse effects on cognition, they may operate through partly distinct mechanisms. By reducing opportunities for interpersonal contact, social isolation may decrease exposure to cognitively stimulating experiences. According to the cognitive reserve hypothesis (Stern, 2002), stimulating experiences across the lifespan contribute to the development of neural and cognitive resources that help individuals cope more effectively with age-related brain changes (Evans et al., 2019; Rodriguez et al., 2017; Stern, 2012). From this perspective, communication during social interaction constitutes a particularly rich form of cognitive stimulation, as it requires the coordination of multiple processes, including lexical retrieval, semantic access, pragmatic inference, turn-taking, and discourse organization (Ybarra et al., 2008). Reduced social contact may therefore limit the repeated activation and reinforcement of these mechanisms over time.

A complementary account is provided by the “use-it-or-lose-it” hypothesis, which proposes that activation and frequent activation and practice help maintain cognitive abilities, whereas reduced engagement may accelerate functional decline (Cardona and Andrés, 2023; Hultsch et al., 1999). In line with this view, the literature on social engagement suggests that active participation in social relationships and activities is beneficial for cognitive functioning by providing sustained cognitive stimulation and opportunities for communicative interaction (Kelly et al., 2017; Penninkilampi et al., 2018).

Overall, both L/SI contribute to cognitive decline and have been identified as potentially modifiable risk factors for cognitive decline and dementia (Evans et al., 2019; Lee et al., 2025; Samtani et al., 2022). It is also important to note that individuals who experience both L/SI appear to show the most pronounced trajectories of cognitive decline, suggesting a cumulative effect of these two phenomena (Kang et al., 2024; Lampraki et al., 2025). Existing evidence also suggests that these associations may be bidirectional (Ellwardt et al., 2013; Okely and Deary, 2019; Yin et al., 2019; Zhong et al., 2017). Declines in cognitive functioning, or the presence of underlying neuropathology, may reduce social connection by making it more difficult for individuals to initiate, maintain, or benefit from social relationships. Over time, these difficulties may contribute to behavioral changes that increase the risk of both L/SI (Greenwood and Smith, 2016).

Against this backdrop, language is particularly relevant to the study of L/SI because it is both a medium of social participation and a potential marker of social disconnection. It is one of the primary mechanisms through which social relationships are established and maintained requiring the dynamic integration of linguistic and cognitive processes such as lexical retrieval, semantic processing, discourse organization, multimodal cues, and reciprocal feedback between interlocutors (Holler and Kuhlen, 2026; Mueller et al., 2018). Everyday social interactions depend on both efficient language production and comprehension, and successful communication requires speakers and listeners to continuously coordinate linguistic, cognitive, and social information in real time (Holler and Kuhlen, 2026). Consequently, L/SI may contribute to poor language functioning through reduced social and intellectual stimulation, and communication difficulties may also hinder successful interactions and relationship maintenance, thereby reinforcing L/SI (Boss et al., 2015; Cachón-Alonso et al., 2023; Cardona and Andrés, 2023; MacDonald, 2017). Nevertheless, despite the centrality of language to social life, relatively few studies have examined language specifically in relation to L/SI. Because social interactions rely on linguistic expression, access to lexical-semantic networks, and pragmatic skills (Dooley and Walshe, 2019; McDonald et al., 2021), patterns of social participation may both shape and reflect language use in aging (Bourassa et al., 2017; Brown et al., 2012; Rehan and Phillips, 2025).

Furthermore, age-related changes, even in healthy aging, may increase vulnerability to L/SI, although these changes do not affect all linguistic processes uniformly. Aging-related effects appear to be particularly pronounced in domains that rely on processing speed, executive control, and working memory (Burke and Shafto, 2004; Chen et al., 2026; Kemper and Sumner, 2001; Shafto and Tyler, 2014). Processes such as lexical retrieval and syntactic processing seem especially vulnerable, with evidence of longer naming latencies, reduced verbal fluency, and a higher incidence of “tip-of-the-tongue” (TOT) phenomena (Burke and Shafto, 2004; Gollan et al., 2008; Ouyang et al., 2020). TOTs are generally thought to arise from weakened connections between semantic and phonological representations, even when word meaning remains intact (Burke and Shafto, 2004; Xie and Wang, 2026). Age-related changes in syntactic processing, are also reflected in reduced grammatical complexity, less frequent use of subordinate structures, and greater difficulty in comprehending syntactically complex sentence (Chan et al., 2001; Kemper and Sumner, 2001; Tyler et al., 2011). Older adults may also show more dysfluencies, anomia, and verbosity in spontaneous speech (Mortensen et al., 2006).

In contrast, vocabulary knowledge and semantic representations remain relatively resistant to normal aging and may even improve over the course of a lifetime, constituting an important protective resource (Burke and Shafto, 2004; Lacombe et al., 2015; Shafto and Tyler, 2014; Verhaeghen, 2003), even if access to this knowledge becomes progressively less efficient (Li et al., 2001). Language comprehension also becomes increasingly dependent on top-down compensatory mechanisms and contextual information (Davis and Johnsrude, 2007; Stine-Morrow et al., 2006), especially when perceptual or cognitive demands increase (Chen et al., 2026; Kroll et al., 2015). However, most studies investigating L/SI and language associations in older adults have focused on verbal fluency, offering only a limited view of broader linguistic abilities. Even within verbal fluency, findings are not fully consistent: some studies report that loneliness predicts faster decline specifically in semantic verbal fluency (Lara et al., 2019a; Simpson et al., 2019), whereas others find no association between them (Windsor et al., 2020).

Against this background, more recent work in computational linguistics and Natural Language Processing (NLP) has begun to broaden the assessment of language by analyzing spontaneous or semi-structured speech produced in more naturalistic communicative contexts (Asgari et al., 2017; Mueller et al., 2018). These approaches have been proposed as more ecologically valid methods for capturing subtle changes that may not be fully detected by traditional neuropsychological testing (Gagliardi, 2024; Wang et al., 2024; Yamada et al., 2021). Features such as reduced syntactic complexity, longer pauses, reduced pitch variability, increased filler usage (e.g., “um,” “well”), and pronoun usage patterns have been identified as markers of cognitive decline and loneliness (Asgari et al., 2017; Badal et al., 2021a, 2021b; Wang et al., 2024). For example, Yamada et al. (2021) observed fewer inflections, longer pauses, more filler words, and fewer positive words in older adults who reported higher loneliness scores.

Despite this growing body of research, the association between language and L/SI remains difficult to synthesize due to substantial methodological heterogeneity, including differences in how both language and social disconnection are conceptualized and measured. Language outcomes range from brief single-task indicators, especially verbal fluency, to complex discourse-based measures and computational analyses of naturalistic speech (Boss et al., 2015; Evans et al., 2019; Harrington et al., 2023). Moreover, language is often treated as a proxy for global cognition rather than an independent domain of interest (Simpson et al., 2019; Stafford et al., 2024). This makes it difficult to determine which aspects of language are most closely linked to loneliness, social isolation, or broader social connection.

To address this gap, the present scoping review maps how language has been examined in the specific context of L/SI in older adults. For the purposes of this work, language functioning is conceptualized as a multidimensional communicative phenotype rather than as a single cognitive domain, encompassing linguistic expression, lexical-semantic access, discourse organization, and communicative use in social interaction (Dooley and Walshe, 2019; Mueller et al., 2018). This includes traditional language-related neuropsychological tasks, such as verbal fluency and naming, but also higher-level discourse measures, semantic-processing tasks, and speech-derived markers obtained from naturalistic or semi-structured communication. Traditional tasks provide standardized indices of specific linguistic and cognitive operations, whereas discourse and speech-based measures capture how these abilities are deployed in communicative contexts that are directly relevant to L/SI. Moreover, traditional measures often fail to capture the functional integration of cognitive and linguistic skills required for real-world social participation (MacDonald, 2017). Integrating traditional neuropsychological tasks with conversational, discourse-based, and computational markers may therefore provide a more comprehensive and ecologically valid account of language functioning in socially disconnected older adults. By adopting this multidimensional approach to language and communication, it will allow a better understanding of the association between L/SI and language, the review aims to clarify how different aspects of linguistic functioning are associated with L/SI and to identify which dimensions of language have been most frequently overlooked. Specifically, this scoping review aims to: (1) identify the psycholinguistic and linguistic domains most frequently examined in relation to L/SI in older adults; (2) characterize the range of linguistic measures, tasks, and computational approaches used across studies; and (3) to synthesize evidence regarding the relation between linguistic functioning and L/SI. Thus, this review seeks to clarify how language has been considered in research on L/SI experiences in aging, identify methodological and conceptual gaps, and highlight directions for future research.

## 2 Materials and methods

The current scoping review was reported in accordance with the Preferred Reporting Items for Systematic Reviews and Meta-Analyses 2020 (PRISMA 2020; Page et al., 2021) and the Preferred Reporting Items for Systematic Reviews and Meta-Analyses extension for Scoping Reviews (PRISMA-ScR, Tricco et al., 2018).

### 2.1 Literature search strategy

Three databases were used to identify potential records: PubMed, Scopus, and Web of Science (collections: Web of Science Core Collection, KCI-Korean Journal Database, Medline, Research Commons, SciELO Citation Index). Records were searched from inception until the 12th January 2026 by combining search terms related to aging (e.g., aging, elder, older), language (e.g., speech, language, linguistic, verbal), and L/SI (e.g., loneliness, lonely, social isolation). The full set of expressions used in the literature search of each database, as well as additional document-type exclusion filters, can be found in Table S1 of the Supplementary Materials section. Additionally, we performed citation searching in the records that were submitted to full-text reading as a way to identify relevant studies that were missed by the initial database search.

### 2.2 Eligibility criteria

The eligibility criteria were structured using the Population, Concept, Context (PCC) framework (Peters et al., 2020), a commonly recommended approach to studies selection in scoping reviews.

Regarding population, studies should include a sample of participants aged 60 years or older, regardless of clinical status. Works that met all other eligibility criteria and focused on aging, even if they included a broader sample (e.g., participants ≥ 50 years), as long as the mean age was ≥60 years, or a stratified sample, provided it included a group of older adults, were also eligible. Exceptions were also made for longitudinal studies, as in if the sample had a mean age of ≥60 years at follow-up, even if it was younger at baseline. This approach ensured the inclusion of studies relevant to later-life language functioning while allowing for flexibility in study designs.

The concept of the present review was to explore the association between language functioning and L/SI and to map the linguistic tasks and measures used in this literature. As such, eligible studies were required to include at least one measure of loneliness or social isolation, and one measure of linguistic processing. For social isolation measures, we considered the recommendations of Gardam et al. (2023) as criteria. Studies that included measures intended to assess SI but were not explicitly identified by Gardam et al. (2023) were subjected to full-text screening for further analysis. Regarding loneliness, we also considered measures designed for perceived/subjective social isolation, since these terms are used interchangeably across the literature. As for language-related measures, for the purposes of this review, this category was defined broadly to include classic language tasks (e.g., verbal fluency, lexical retrieval, naming, discourse production, and semantic processing), as well as speech-derived linguistic markers obtained through computational analyses when they indexed language use in communication. Because some of these measures, especially verbal fluency and computational markers, also draw on broader executive or cognitive processes, they were treated here as language-relevant rather than as pure language measures.

More perceptual or auditory-based language measures were excluded when the primary outcome concerned auditory speech recognition or speech perception under degraded listening conditions, such as speech-in-noise tasks. Although these tasks engage linguistic and cognitive mechanisms, performance is also strongly influenced by peripheral hearing acuity, auditory temporal processing, and the acoustic characteristics of the listening environment. In older adults, age-related hearing loss and changes in auditory processing are major determinants of performance on such tasks, making it difficult to disentangle linguistic difficulties from sensory constraints. Because the primary aim of this review was to examine the relationship between L/SI in older adults, and to reduce conceptual and methodological heterogeneity, studies in which performance was heavily driven by auditory-perceptual demands were considered outside the scope of the review—for further discussion of the associations between hearing impairment, loneliness, social isolation, and cognition see Maharani et al. (2019) and Shukla et al. (2020).

Additionally, global cognitive functioning measures (e.g., Mini-Mental State Examination [MMSE], Folstein et al., 1975; Montreal Cognitive Assessment [MoCA], Nasreddine et al., 2005) were only considered eligible when analyses examined domain-specific scores, rather than only a global score, allowing the extraction of language-related outcomes. Finally, eligible studies were required to include statistical analyses explicitly examining the relationship between language measures and L/SI (e.g., correlation analyses, regression models, mediation/moderation analyses). Studies that included both constructs but did not analyze their relationship were not considered eligible.

The context is aging focused studies, including community, clinical, and population-based studies across different settings. This review included empirical studies reporting original data, regardless of whether they used observational (e.g., cross-sectional, longitudinal, or correlational) or interventional designs. Records not reporting original complete empirical studies (e.g., conference proceedings, conference abstracts, book chapters, study protocols, systematic reviews) were not included. Non-peer-reviewed records, such as dissertations and preprints, were also excluded. No language restrictions were applied, as automatic translation tools could be used when necessary.

### 2.3 Study selection procedure

After removing duplicates, the records retrieved from the database searches were screened according to the eligibility criteria in two successive stages. The first involved title and abstract screening, and the second a full-text review. Both stages were conducted independently by the two authors, DP and MA. In cases of disagreement, the raters discussed the study to achieve consensus.

### 2.4 Data extraction and synthesis

A narrative synthesis approach was adopted to organize and interpret the results of this review. This decision was based on the expected paucity of studies specifically examining language in relation to L/SI, together with substantial heterogeneity in study populations, L/SI constructs, language measures, analytic strategies, and covariate adjustment. Under these conditions, a meta-analysis was not considered appropriate. Instead, the aim was to map the field, identify recurring patterns, and highlight methodological gaps and future directions. Accordingly, a scoping review with narrative synthesis was considered the most suitable approach (Munn et al., 2018).

For each study included in the scoping review, we summarized information regarding: (a) study identification (first author’s last name, year of publication), (b) participant characteristics (sample size, percentage of female participants, mean age, age range) and country/ies associated with data collection), (c) study design, (d) L/SI measures, (e) language measures, and (f) the main findings exploring the relationship between language and L/SI outcomes (see Table 1).

**Table 1:** Summary of the studies included in the scoping review organized by methodological approaches and linguistic processing domains.

Study	Sample (sex; mean age; age range)	Study design	L/SI outcome(s)	Language outcome(s)	Main findings
Language production (more traditional tasks and manual approaches)					
Arbuckle et al. (2004)	142 (71.1% F; M_F_ = 75.22, M_M_ = 74.12; 63–95), community-dwelling older adults, Canada	Longitudinal (mean 19-month interval; 4–26)	Social Support Questionnaire 20-item UCLA Loneliness Scale Social network/context assessment	Off-Target Verbosity (OTV) measured during a life history questionnaire	High verbosity was indirectly linked to loneliness via reduced competence; when controlling for competence, higher verbosity predicted lower loneliness. Off-target verbosity was weakly and non-significantly correlated with UCLA loneliness. Path analysis showed no significant direct relationship between OTV and loneliness in the final longitudinal model.
Barnett et al. (2023)	142 total, USA: YAG: 62 (62.9% F; *M* = 20.69; 18–28). OAG: 80 (65.0% F, *M* = 76.54; 60–98).	Cross-sectional; Comparison between age cohorts	3-item Loneliness Scale (TILS)	Speech quality in verbal description tasks (tangentiality, quantity, and egocentrism)	Regression models indicated that greater tangentiality and lower speech quantity predicted higher loneliness in older adults.
Beller and Wagner (2020)	40,797 (57% F; *M* = 68; 50+), 14 European countries	Longitudinal; SHARE Waves 5–6	3-item Revised UCLA Loneliness Scale	Semantic fluency (AFT)	Loneliness was negatively associated with verbal fluency. Loneliness significantly predicted a decline in verbal fluency over time. The association was moderated by cultural individualism, being stronger in collectivistic countries.
Bethell et al. (2024)	27,765 (50.9% F; *M_F_* = 65.44, *M_M_* = 65.78; 45–85), Canada	Cross-sectional; CLSA	3-item Revised UCLA Loneliness Scale Social isolation index (0–5; living arrangements, friends/neighbors, relatives/siblings, children, social participation)	Semantic fluency (AFT) Phonemic fluency (COWAT)	Loneliness was inversely associated with semantic and phonemic fluency in unadjusted models. In models adjusted for age, education, and hearing, loneliness remained a robust direct predictor of poorer performance in both fluency tasks. Social isolation also showed significant direct effects on COWAT. The same was observed for AFT but only in male participants. Both loneliness and social isolation significantly mediated the association between neuroticism and fluency outcomes.
Carbone et al. (2022)	113 (69.0% F; M ≈ 83.4, 61–94), Italy, Mild Dementia: 58 (38\u00B0F; *M* = 79.50) Moderate Dementia: 55 (40\u00B0F; *M* = 87.18)	Cross-sectional	6-item Social and Emotional Loneliness Scale (includes emotional and social subscales).	Narrative Language Test (NLT; picture and sequence description).	In moderate dementia, loneliness explained 8% of variance in language skills. Social loneliness specifically accounted for 18% of variance in this group. In mild dementia, social loneliness predicted NLT performance.
DiNapoli et al. (2014)	267 (64.8% F; *M* = 78.50; 70–94), USA	Cross-sectional	Perceived isolation subscale of the LSNS-6 (4 items) Social disconnectedness subscale of the LSNS-6 (2 items: network size and contact frequency).	Boston Naming Test Semantic fluency (AFT) Phonemic fluency (COWAT)	Social isolation was significantly associated with language abilities in unadjusted models. However, this association became non-significant after controlling for age, education, and other covariates.
Domenicucci et al. (2025)	115 (65.2% F; *M* ≈ 82; 61–94), mild to moderate dementia, Italy CST group: 68 (*M* = 81.67, 61–94) Active control group: 47 (*M* = 82.64, 62–94)	RCT with pre-, post-, and 3-month follow-up	6-item de Jong Gierveld Loneliness Scale	Narrative Language Test (NLT)	Baseline social loneliness and emotional loneliness were negatively associated with NLT scores. Baseline loneliness did not significantly predict long-term linguistic gains following CST intervention.
Estrella et al. (2024)	2,155 (53.7% F; *M* = 55.90; 45+), U. S. Hispanics/Latinos	Longitudinal; HCHS/SOL SCAS and SOL-INCA (7-year follow-up)	3-item Revised UCLA Loneliness Scale Social Network Embeddedness Index	Phonemic fluency (FA)	Baseline loneliness did not predict change in word fluency in fully adjusted models, despite prior cross-sectional associations with lower baseline fluency. Similarly, social isolation was not a significant predictor of 7-year change in word fluency after adjustment for sociodemographic and depressive variables.
Gilmour (2011)	13,176 (n/a F; *M* = n/a; 65+), household population, Canada	Cross-sectional; 2009 CCHS	3-item loneliness measure (companionship, isolation, feeling left out) SI indicators: Living arrangement (living with partner) and frequent social participation (weekly activities)	Semantic fluency (AFT)	Loneliness was significantly associated with low cognitive performance in the semantic fluency task, but it did not persist in multivariate models. Participants with low scores were less likely to live with a partner compared to those with moderate/high performance. No significant association was found between the frequency of participation and semantic fluency.
Huang et al. (2025)	24,615 (56.6% F; *M* = 64.90; 50–90), 29 European countries	Longitudinal; SHARE Waves 5–9	3-item Revised UCLA Loneliness Scale	Semantic fluency (AFT)	Loneliness partially mediated the relationship between disadvantaged childhood socioeconomic position and late-life verbal fluency.
Kyaw and Levine (2023)	541 (64.7% F; *M* = 70.90, 49+), diagnosis of dementia, South Africa	Cross-sectional; HAALSI (2019–2020)	CES-D single item (“During the past week, how often have ‘I felt lonely”)	Semantic fluency (AFT) Naming (BNT)	Low levels of loneliness were associated with greater global cognitive function but was not significantly associated with semantic fluency in fully adjusted models.
Lampraki et al. (2025)	33,741 (57.1% F; *M* = 61.40; 50–99), 12 European countries	Longitudinal; SHARE Waves 1, 2, 4–9	3-item Revised UCLA Loneliness Scale Binary indicator based on the Social Connectedness Scale (network size, proximity, frequency of contact, support, and diversity)	Semantic fluency (AFT)	Compared to the non-isolated/non-lonely profile, both non-isolated/lonely and isolated/lonely individuals showed significantly lower fluency scores, with the latter exhibiting the largest impairment.
Lara et al. (2019a)	1,691 (52.8% F; *M* = 64.50; 50+), Spain	Longitudinal (3-year follow-up); COURAGE and Edad Con Salud	3-item Revised UCLA Loneliness Scale SI Index (0–5; living alone/unmarried; < monthly contact with children, family, and friends; no organization participation)	Semantic fluency (AFT)	Both loneliness and social isolation were significantly associated with lower verbal fluency scores in adjusted models. No statistically significant verbal fluency and loneliness/social isolation association over time.
Lee et al. (2024)	785 (62.8% F; *M* = 67.30; n/a), Republic of Korea ≥ 65 group: 513 (*M* = 71) < 65 group: 272 (*M* = 60.40)	Cross-sectional; KoGES cohort	UCLA Loneliness Scale (cutoff ≥ 44) and CES-D single item	Korean-Boston Naming Test (from SNSB-C)	Loneliness was associated with a 2-fold increase in the odds of global cognitive impairment, but the specific association with the language domain score was non-significant in multivariate models.
Li et al. (2025)*	43 (69.8% F; *M* = 66; n/a), Taiwan	Cross-sectional; ERP study	20-item UCLA Loneliness Scale	Semantic verbal fluency	No significant correlation found between loneliness scores and CVF scores.
Luchetti et al. (2022)	24,689 opposite-sex couples (*M_F_* = 63.20; *M_M_* = 66.30; 50+), 28 countries (European + Israel)	Longitudinal dyadic; SHARE Waves 4–6, and 8	3-item Revised UCLA Loneliness Scale	Semantic fluency (AFT)	Significant actor effects of loneliness on verbal fluency were observed for both males and females, indicating that higher own loneliness was associated with poorer fluency. Significant partner effects were also found, suggesting that a partner’s loneliness predicted lower verbal fluency independently of one’s own loneliness. No sex or age moderation were found.
Neff et al. (2024)*	83 (n/a; n/a; 65–91), community-dwelling older adults, Switzerland	Cross-sectional; Ambulatory assessment with Elastic Net regression (MOASIS project).	3-item revised UCLA loneliness scale; SI indicators (civil status – no partner; naturalistic speech length).	Semantic fluency (RWT) Phonemic fluency (LPS6)	Semantic fluency ability positively predicted naturalistic speech length.
Okely and Deary (2019)	767 (48.2% F; *M* = n/a; 73–79) community dwelling older adults, Scotland	Longitudinal; LBC1936 Waves 2–4; age comparison	Single-item loneliness (“How much of the time during the past week have you felt lonely?”) SI indicator: Marital status	Crystallized ability score (phonemic fluency, NART, WTAR)	Higher crystallized ability at age 73 predicted lower loneliness at age 76. Cross-sectionally, loneliness and crystallized ability were negatively correlated only at 76, but not at age 73 or age 79. Loneliness did not predict later crystallized ability.
O’Luanaigh et al. (2012)	466 (56.6% F; *M* = 75.45; n/a) community-dwelling older adults, Ireland	Cross-sectional; Dublin Healthy Ageing study	CES-D single item (“Do you feel lonely?”) Wenger’s Social Networks Typology	Semantic fluency (AFT) Phonemic fluency (FAS)	Loneliness was associated with reduced performance in animal naming. No statistically significant relationship was found between loneliness and letter fluency.
Rehan and Phillips, 2025	126 (40% F; *M* = 71.50; 50–90), with Mild Cognitive Impairment, Canada	Cross-sectional; COMPASS-ND	Single item loneliness (“Do you feel lonely, or do you feel very lonely?”) SI indicators (living arrangements, frequency of contact with friends/relatives, self-rated social participation, and social participation index)	Semantic and Phonemic fluency (from D-KEFS).	Lower social participation was associated with poorer verbal fluency performance: participants with low social scored lower than those with normal and high participation. Loneliness and perceived social support were not significantly associated with verbal fluency.
Rodriguez et al. (2017)	2,339 (53.3% F; *M* = n/a; 65–79), retired individuals, including people with Mild Neurocognitive Disorder, Germany (it is a subsample of the study).	Cross-sectional; LIFE-Adult-Study	LSNS	Semantic fluency (AFT; CERAD-plus test)	Higher social isolation was associated with poorer verbal fluency in fully adjusted models.
Schnittger et al. (2012)	579 (69% F; *M* = n/a; n/a), community-dwelling older adults, Ireland	Cross-sectional	Jong-Gierveld Loneliness Scale LSNS	Semantic fluency (AFT) Naming task (from CAMCOG)	Animal naming emerged as risk factor for social but not emotional loneliness. Animal naming did not mediate the effect of social support on social loneliness. No statistically significant association between animal naming and social support was found.
Shankar et al. (2013)	6,034 (54.7% F; *M* = 65.60; 50+), England	Longitudinal; ELSA, wave 2 and 4 (4-year follow-up)	3-item Revised UCLA Loneliness Scale SI Index (0–5; living alone/unmarried; <monthly contact with children, family, and friends; no organization participation)	Semantic fluency (AFT)	Both social isolation and loneliness were significantly associated with poorer verbal fluency. Longitudinally, higher baseline social isolation predicted lower verbal fluency scores after 4 years, whereas loneliness was not significantly associated with change in verbal fluency over time. No statistically significant interaction between isolation and loneliness was observed for verbal fluency.
Simpson et al. (2019)	962 (52.3% F; *M* = 64.30; 50–98) community-dwelling adults, Spain	Cross-sectional (ELES pilot)	6-item de Jong Gierveld Loneliness Scale	Semantic fluency (AFT) Phonemic fluency(/s/)	Loneliness was a significant predictor of semantic fluency but not phonological fluency. The association between loneliness and semantic fluency was moderated by age, with mild loneliness showing smaller age-related declines in older participants, and severe loneliness associated with increasing declines in semantic fluency with advancing age. Education and cognitive function were protective for both types of verbal fluency, and females showed higher phonological, but not semantic, fluency after adjustment.
Stokes et al. (2023)	2,122 (Opposite-sex couples; *M_F_* = 67.50; *M_M_* = 71.60; 65+), USA	Longitudinal; HRS (2010–2020 waves)	11-item abbreviated Revised UCLA Loneliness Scale	Semantic fluency (AFT)	Loneliness predicted own and partner’s loneliness, but baseline loneliness was not significantly associated with verbal fluency at follow-up.
Wang et al. (2025)	320 (n/a; n/a; 65+), Japan (simulated sample)	Cross-sectional; Simulated dataset based on JSTAR and SHARE parameters	3-item Japanese version of UCLA Loneliness Scale	Fluency Narrative complexity	Found a strong inverse relationship between narrative complexity and loneliness. Depressive symptoms were found to mediate the association between language impairment and loneliness.
Wilson et al. (2007)	823 (75.7% F; *M* = 80.70; n/a), USA Without AD group: 716 (77.2% F; *M* = 80.30; n/a) With AD group: 76 (61.8% F; *M* = 85.10; n/a)	Longitudinal (4-year follow-up); Rush Memory and Aging Project	Modified version of de Jong Gierveld Loneliness Scale (only emotional loneliness) SI indicators: number of children, family, friends, and frequency of interactions; average item score of six items associated with social interaction (e.g., visiting a relative or friend)	Semantic memory composite (fluency, BNT, NART)	At baseline, emotional loneliness was associated with impaired cognitive functioning, including in the semantic memory domain, after controlling for sex, age, and level of educational attainment. With this baseline effect controlled for, loneliness was associated with a more rapid decline in global cognition and semantic memory composite scores.
Windsor et al. (2020)	516 (50.0% F; *M_F_* = 85.10, *M_M_* = 84.70; 70–100), Germany	Longitudinal (13-year); BASE	8 items from the Revised UCLA Loneliness Scale (4 emotional, 4 social subscales) Network size (map procedure) and prosocial activity index (sum of 6 support types)	Semantic fluency (AFT)	Larger social networks predicted higher baseline fluency. Low education combined with high social loneliness predicted steeper decline. Larger social networks were independently associated with overall better verbal fluency performance, though loneliness was not a consistent predictor.
Yin et al. (2019)	5,885 (55.4% F; *M* = 65.30; 50+), England	Longitudinal; ELSA (10-year follow-up)	3-item Revised UCLA Loneliness Scale	Semantic fluency (AFT)	Baseline loneliness predicted steeper fluency decline. Fluency decline also predicted increases in loneliness (bidirectional association). Baseline loneliness was negatively associated with baseline verbal fluency and predicted a steeper 10-year decline.
Zhou et al. (2022)*	88 (total), Taiwan YAG: 38 (*M* = 22.21) OAG: 50 (72% F; *M* = 66.38; 60–80) community-dwelling older adults	Cross-sectional	20-item UCLA Loneliness Scale	Category verbal fluency Vocabulary (WAIS-III)	Loneliness was not significantly associated with category verbal fluency or vocabulary scores; these verbal abilities did not predict word recognition speed
Discourse production (Computational and automatic approaches)					
Badal et al. (2021a)	80 (63.8% F; *M_F_* = 81.60, *M_M_* = 85.50; 66–94) independent-living older adults, USA	Cross-sectional; Qualitative and ML analysis; NLP of semi-structured interviews	3-item UCLA Loneliness Scale Qualitative interview question (“Do you ever feel lonely?”)	NLP-derived speech features (sentiment, emotion, and response length)	ML models predicted qualitative loneliness with 94% precision based on speech features. Lonely individuals provided longer responses to direct questions and expressed significantly greater sadness.
Badal et al. (2021b)	97 (64.9% F; *M_F_* = 81.70, *M_M_* = 86.20; 66–94) independent-living older adults, USA	Cross-sectional; Qualitative and ML analysis; NLP of semi-structured interviews	3-item Revised UCLA Loneliness Scale. NLP-derived extraction of social contacts from interview transcripts	NLP features (lexical complexity, sentence structure, and pronoun density)	First-person plural pronoun density was negatively associated with loneliness in women. Higher loneliness was further linked to lower sentence complexity and shorter response lengths.
Neff et al. (2024)*	83 (n/a; n/a; 65–91), community-dwelling older adults, Switzerland	Cross-sectional; Ambulatory assessment with Elastic Net regression (MOASIS project).	3-item Revised UCLA Loneliness Scale; SI indicators (civil status – no partner; naturalistic speech length).	Naturalistic speech length	Bivariate correlations between social loneliness and speech length were non-significant in network analysis. “No partner” status was the strongest predictor of reduced naturalistic speech length.
Wang et al. (2024)	97 (64.9% F; *M* = 83.20; 65–94), USA	Cross-sectional; XAI + LIWC-22; Transformer-based ML classification models of semi-structured interviews	20-item UCLA Loneliness Scale (v3)	NLP-derived LIWC-22 linguistic features from semi-structured interviews (themes, fillers, pronouns)	Social themes and pronoun usage were identified by XAI as the most important linguistic features for classifying loneliness from unstructured speech (accuracy = 0.889).
Yamada et al. (2021)	57 (52.6% F; *M* = 73.20; 62–81), Japan	Cross-sectional; speech elicitation; ML regression and binary-classification models using 10-fold cross-validation	20-item Japanese version of UCLA Loneliness Scale (v3)	NLP speech features (acoustic, prosodic, and linguistic aspects)	Loneliness correlated with multiple speech features and ML models predicted high loneliness with 95.6% accuracy. Increasing loneliness correlated with more filler words and fewer positive words in responses.
Language comprehension and semantic integration					
Li et al. (2025)*	43 (69.8% F; *M* = 66; n/a), Taiwan	Cross-sectional; ERP study	20-item UCLA Loneliness Scale	N400 ERP	Loneliness was moderately negatively correlated with the low-typicality category N400 effect. Lonely individuals exhibited attenuated and delayed neural responses during semantic memory retrieval.
Zhou et al. (2022)*	88 (total), Taiwan YAG: 38 (*M* = 22.21) OAG: 50 (72% F; *M* = 66.38; 60–80) community-dwelling older adults	Cross-sectional	20-item UCLA Loneliness Scale	Lexical ambiguity resolution (lexical decision task)	Perceived loneliness modulated the efficacy of sublexical ambiguity resolution: participants with higher loneliness scores displayed a greater sublexical ambiguity disadvantage, but not general word recognition speed.

* Studies that appear in more than one category; AD = Alzheimer’s disease; AFT = animal fluency task; BASE = Berlin aging study; BNT = Boston naming test; CAMCOG = Cambridge cognitive examination; CCHS = Canadian community health survey; CES-D = center for epidemiologic studies-depression scale; CERAD = consortium to establish a registry for Alzheimer’s disease; CLSA = Canadian longitudinal study on aging; COMPASS-ND = the comprehensive assessment of neurodegeneration and dementia [observational cohort study of The Canadian Consortium on Neurodegeneration in Aging (CCNA)]; COURAGE = Collaborative Research on Ageing in Europe; COWAT = controlled oral word association test; Edad Con Salud = the Spanish longitudinal study on ageing and health; ELES = El Estudio Longitudinal Envejecer en España (The Longitudinal Study of Aging in Spain); D-KEFS = Delis-Kaplan executive function system; ELSA = English longitudinal study of ageing; ERP = event-related potential; F = Female; HAALSI = health and aging in Africa: a longitudinal study of an INDEPTH Community in South Africa; HCHS/SOL = Hispanic community health study/study of Latinos; HRS = health and retirement study; JSTAR = Japanese study on aging and retirement; KoGES = Korean genome and epidemiology study; LIFE-adult-study = adult study’ of the Leipzig Research Centre for Civilization Diseases; LBC1936 = Lothian birth cohort 1936; LSNS = Lubben social network scale; ML = machine learning; MOASIS = mobility, activity, and social interactions Study; n/a = not available/applicable; NART = national adult reading test; NLP = natural language processing; NLT = narrative language test; OAG = older adults’ group; OTV = off-target verbosity; SCAS = sociocultural ancillary study (ancillary to HCHS/SOL); SHARE = survey of health, ageing, and retirement in Europe; SNAC-K = Swedish national study on aging and care in Kungsholmen; SNSB-C = Seoul neuropsychological screening battery; SOL-INCA = study of Latinos-investigation of neurocognitive aging (ancillary to HCHS/SOL); YAG = young adults’ group; WTAR = Wechsler test of adult reading.

## 3 Results

A total of 34 studies were included in the present scoping review. In the following subsections, we first provide an overview of the participants and general characteristics of the included studies, followed by a description of the measures used to assess L/SI. We then summarize the language-related measures employed across studies and finally present the main findings regarding the association between language and L/SI.

### 3.1 Study selection

**Figure 1:**
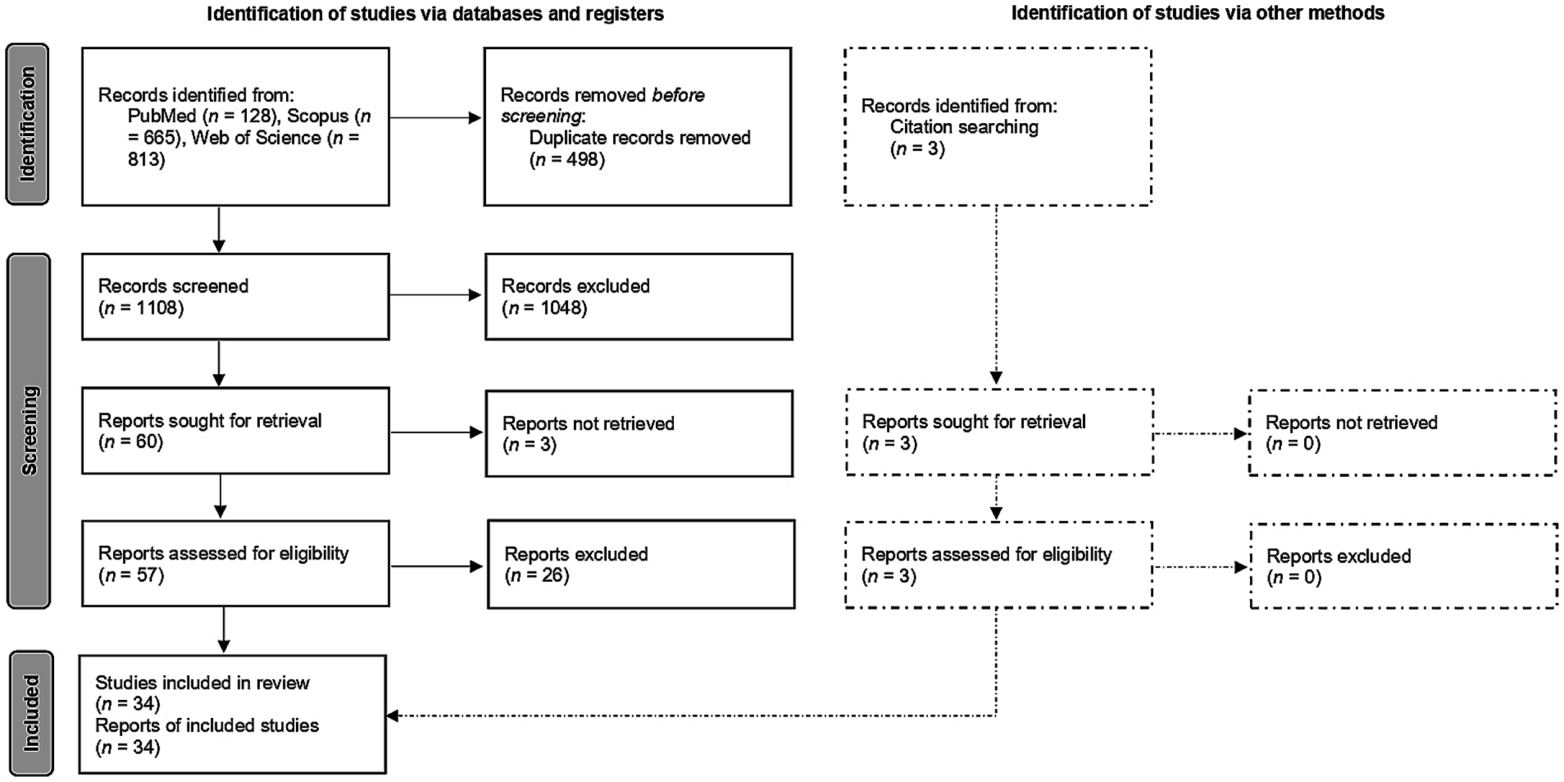
This flow diagram was adapted from the PRISMA (2020) flow diagram template (Page et al., 2021) available used under the Creative Commons Attribution 4.0 International (CC BY 4.0) license.

The database search yielded 1,606 results, and 1,108 after removing duplicates. Of these, after screening titles and abstracts, 60 were selected for full-text review. At this stage, three articles could not be accessed, and 26 were excluded. The main reasons for exclusion were failure to meet article type criteria (i.e., non-peer reviewed articles; *n* = 9), failure to meet sample requirements (*n* = 1), failure to include at least one measure of L/SI (*n* = 2) or one measure of language (*n* = 8), and failure to explore any type of association between the measures in the results (*n* = 6). From citation searching in the records 3 additional studies were included. A total of 34 studies were considered eligible. Interrater reliability, calculated using Cohen’s kappa statistic, was 0.87, indicating a strong level of agreement between raters (McHugh, 2012). A summary of the selection procedures adopted in this scoping review is presented in the PRISMA flow diagram (see Figure 1).

### 3.2 Characteristics of included studies

The studies comprised in this review present a wide range of characteristics, including geographical distribution, sample size, participant characteristics, and study design, including large-scale longitudinal cohort analyses, cross-sectional observational studies, naturalistic speech analyses, and machine-learning-based approaches to linguistic data.

Starting with the studies’ publication year, it ranges from 2004 to 2025, with most studies (*n* = 26) being published between 2019 and 2025. Of these, almost half are from 2024 and 2025, which shows an increasing relevance of the field.

The geographical representation of the studies was diverse but showed a clear predominance in industrialized Western countries, complemented by studies conducted in Asian and African contexts. Europe was the most studied region, largely driven by the wide use of large datasets such as the Survey of Health, Aging and Retirement in Europe (SHARE), which includes participants from multiple European countries and Israel (e.g., Beller and Wagner, 2020; Huang et al., 2025; Lampraki et al., 2025; Luchetti et al., 2022). Additional European studies were conducted using national or regional cohorts in countries such as the United Kingdom, (e.g., Okely and Deary, 2019; Shankar et al., 2013; Yin et al., 2019), Germany (e.g., Rodriguez et al., 2017; Windsor et al., 2020), Spain (e.g., Lara et al., 2019a; Simpson et al., 2019), Italy (e.g., Carbone et al., 2022; Domenicucci et al., 2025), Ireland (O’Luanaigh et al., 2012; Schnittger et al., 2012), and Switzerland (Neff et al., 2024). North American research was primarily based in Canada and in the United States, with studies drawing on both national datasets, as Canadian Longitudinal Study on Aging (CLSA) or Health and Retirement Study (HRS; e.g., Bethell et al., 2024; Gilmour, 2011; Rehan and Phillips, 2025; Stokes et al., 2023), and regional samples from community and residential settings in Canada (Arbuckle et al., 2004) and in the United States (Badal et al., 2021a, 2021b; Barnett et al., 2023; DiNapoli et al., 2014; Estrella et al., 2024; Wang et al., 2024; Wilson et al., 2007). Asian studies were conducted in Japan (Wang et al., 2025; Yamada et al., 2021), South Korea (Lee et al., 2024), and Taiwan (Li et al., 2025; Zhou et al., 2022). And, lastly, a study from South Africa examined older adults living in a rural community as part of the research project Health and Aging in Africa: A Longitudinal Study of an INDEPTH Community in South Africa (HAALSI; Kyaw and Levine, 2023). Overall, this distribution is geographically diverse but remains heavily concentrated in Western populations, with comparatively fewer studies conducted in low- and middle-income regions.

Smaller samples (<150) were typically seen in neuroscientific or NLP studies (e.g., Li et al., 2025; Yamada et al., 2021), reflecting the more resource-demanding nature of these methodologies. Clinical or specific subsample studies generally included between 100 and 800 participants (e.g., Okely and Deary, 2019; Rehan and Phillips, 2025), while national cohort longitudinal studies frequently involved several thousand participants (1,500-10,000; e.g., Lara et al., 2019a; Shankar et al., 2013). Finally, multinational survey studies relied on samples exceeding 20,000 participants (e.g., Bethell et al., 2024; Huang et al., 2025; Lampraki et al., 2025).

Most studies focused on large datasets of community-dwelling older adults aged 50 years or older, such as the SHARE or the English Longitudinal Study of Aging (ELSA; e.g., Beller and Wagner, 2020; Luchetti et al., 2022; Shankar et al., 2013). A smaller subset included clinical groups, particularly individuals with mild cognitive impairment (Rehan and Phillips, 2025) or dementia (e.g., Carbone et al., 2022; Wilson et al., 2007). Because associations observed in clinical samples are not directly comparable to those observed in normative/community aging, findings from these populations should be interpreted cautiously in the overall synthesis.

Research designs varied widely, encompassing large-scale longitudinal studies, cross-sectional analyses, experimental studies, and technology-supported approaches. Most studies were cross-sectional (*n* = 19) and longitudinal (*n* = 13) observational designs. Of these, several included long-term follow-up periods of 10 to 18 years (e.g., Lampraki et al., 2025; Yin et al., 2019). Other adopted medium-term follow-up intervals range from approximately 4 to 8 years (e.g., Estrella et al., 2024; Okely and Deary, 2019) to shorter intervals (e.g., Arbuckle et al., 2004; Shankar et al., 2013).

As for studies using cross-sectional designs, many focused on single waves of large national or international surveys (e.g., Gilmour, 2011; Simpson et al., 2019) or regional and clinical samples (e.g., DiNapoli et al., 2014; Rodriguez et al., 2017). Moreover, some studies implemented comparative designs, contrasting groups by age (e.g., Barnett et al., 2023; Zhou et al., 2022) or clinical severity (Carbone et al., 2022). Experimental and intervention-based approaches were less common (*n* = 2). However, they included a randomized controlled trial (Domenicucci et al., 2025) and an event-related potentials (ERPs) experiment (Li et al., 2025).

### 3.3 Conceptualization and measurement of loneliness and social isolation

Across the included studies, there is a well-established distinction in the literature between subjective experiences of loneliness and objective social disconnection, although operationalizations vary considerably. Considering the primary exposure of interest, we identified four main tendencies regarding the L/SI measures used in the studies: studies focusing on loneliness, on social isolation, on both, and on loneliness and other social connection measures.

Most studies (*n* = 16) assessed loneliness, focusing exclusively on the subjective, distressing perception of social disconnection. They typically employed validated loneliness scales (e.g., different versions of the UCLA Loneliness Scale) and did not incorporate social isolation or other social connection measures (e.g., Barnett et al., 2023; Domenicucci et al., 2025; Lee et al., 2024; Stokes et al., 2023). See Table S2 in of the Supplementary Materials section for a summary of loneliness measures used in the studies.

A small subset of studies (*n* = 3) focused exclusively on social isolation or social activity, without emphasizing subjective loneliness (DiNapoli et al., 2014; Neff et al., 2024; Rodriguez et al., 2017). In DiNapoli et al. (2014) and Rodriguez et al. (2017) studies, the Lubben Social Network Scale (LSNS-6), a shortened version of the LSNS recommended by Gardam et al. (2023), was used to quantify structural disconnection and network engagement. Participants scoring less than 12 were categorized as “socially isolated.” As for Neff et al. (2024), although they did not employ a standardized social isolation instrument, we opted to categorize them in this category, due to the study’s empirical focus on validating “length of own speech” as an objective indicator of everyday social activity.

Five studies assessed both loneliness and social isolation, although none used the measures recommended by Gardam et al. (2023). Schnittger et al. (2012) used the LSNS-18, a different version of the Lubben Social Network Scale from those recommended by Gardam et al. (2023). In the remaining studies, social isolation was often operationalized through composite indices combining marital/cohabitation status, frequency of contact with family and friends, and participation in groups or social activities (Bethell et al., 2024; Lampraki et al., 2025; Lara et al., 2019a; Shankar et al., 2013). Although these measures were not specifically recommended by Gardam et al. (2023), they all incorporated multiple indicators capturing different dimensions of social isolation. Within this category, the study by Lampraki et al. (2025) is particularly notable for adopting a psychosocial profile approach, classifying participants into combined loneliness/isolation profiles such as “isolated but not lonely” and “lonely-in-the-crowd.” This approach of subjective and objective social disconnection as distinct psychosocial risk patterns, treats the co-occurrence and dissociation of subjective and objective social disconnection as distinct psychosocial risk pattern, rather than examining loneliness and social isolation as independent predictors. See Table S3 for a summary of the measures used across studies.

A further group of studies (*n* = 10) assessed loneliness alongside broader social connection indicators that do not constitute formal measures of social isolation, as suggested in Gardam et al. (2023)‘s study, focusing instead on social participation, network resources, or relational quality. For example, Gilmour (2011) and Rehan and Phillips (2025) measured social participation, while Estrella et al. (2024) included single indicators such as marital status. Windsor et al. (2020) and Simpson et al. (2019) assessed network size, satisfaction, or prosocial support. Similarly, Arbuckle et al. (2004) combined structural and functional indicators of social support into a composite factor, capturing network size, satisfaction, and perceived support. Badal et al. (2021b) assessed loneliness and complemented it with measures from the MacArthur Studies of Successful Aging, capturing emotional and instrumental social support as well as negative relationship aspects, which reflect the functional quality of social relationships rather than the structural absence of social ties.

Collectively, there is a lot of overall heterogeneity across studies with a strong focus on subjective loneliness, whereas objectively defined social isolation and broader social connection constructs are less consistently operationalized.

### 3.4 Linguistic domains and language measures

Overall, the linguistic measures captured both language production and comprehension, using a wide range of approaches, that ranged from traditional neuropsychological tests to NLP-based methods. These included verbal fluency, lexical retrieval and naming, discourse production, semantic processing, and computational linguistic markers. Some studies included more than one language domain or combined traditional tasks with computational analyses. To improve conceptual clarity, the measures are described below according to their predominant functional focus: language production and lexical retrieval, discourse production and naturalistic communicative output, and comprehension or semantic-integration measures. This organization should be interpreted pragmatically, since several tasks, especially verbal fluency and computational speech markers, draw on multiple linguistic, cognitive, and communicative processes.

Language production and lexical-retrieval measures were the most frequently represented. The most predominant domain was verbal fluency. In fact, several studies (*n* = 16) only included verbal fluency tasks. Their widespread use likely reflects their ease of administration, brevity, and suitability for large-scale population studies. Of these, 11 assessed only semantic fluency, most commonly animal naming (Beller and Wagner, 2020; Gilmour, 2011; Huang et al., 2025; Lampraki et al., 2025; Lara et al., 2019a; Luchetti et al., 2022; Rodriguez et al., 2017; Shankar et al., 2013; Stokes et al., 2023; Windsor et al., 2020; Yin et al., 2019). One assessed only phonemic fluency (Estrella et al., 2024), where participants generate words beginning with specific letters (e.g., F, A, or S), while four studies included both (Bethell et al., 2024; O’Luanaigh et al., 2012; Rehan and Phillips, 2025; Simpson et al., 2019). Other studies (*n* = 8) used vocabulary and picture-naming tasks, such as the Boston Naming Test (BNT; DiNapoli et al., 2014; Kyaw and Levine, 2023; Lee et al., 2024; Schnittger et al., 2012; Wilson et al., 2007), vocabulary subtests from the WAIS-III (Zhou et al., 2022), reading tests like the National Adult Reading Test (NART) and Wechsler Test of Adult Reading (WTAR; Okely and Deary, 2019; Wilson et al., 2007), or the Multiple Choice Vocabulary Intelligence Test (MWT-B; Neff et al., 2024) to evaluate word retrieval and verbal knowledge.

Discourse production and naturalistic communicative-output measures were less common but conceptually important, as they capture language beyond isolated word-level performance. Some studies (*n* = 5) investigated discourse production, focusing on the structure and informational content of spoken or written language. These assessments included measures such as off-topic verbosity (OTV; Arbuckle et al., 2004; Barnett et al., 2023), narrative coherence (Wang et al., 2025), and informational content derived from structured tasks such as picture description, autobiographical recall, or narrative storytelling using tools like the Narrative Language Test (NLT; Carbone et al., 2022; Domenicucci et al., 2025). Within this broader communicative level, recent works (*n* = 5) introduced computational linguistic markers, automatically extracted from transcribed interviews or speech samples using NLP and related machine-learning tools. Across the included studies, the extracted features covered multiple linguistic and paralinguistic dimensions. Some metrics of interest included syntactic complexity, lexical richness and diversity, pronoun usage patterns (e.g., “I” vs. “we”), speech fluency indicators (e.g., filled pauses or hesitation markers), response length, and the presence of conversational fillers (e.g., Badal et al., 2021b; Wang et al., 2024). Some studies further extended these analyses by incorporating acoustic and prosodic features, such as pitch variation, pause duration, and spectral characteristics of speech (Yamada et al., 2021). Additionally, there were studies also focused on affective and sentiment-related features, such as overall polarity (positive vs. negative tone) and the expression of discrete emotions such as sadness, joy, fear, anger, and disgust (Badal et al., 2021a; Wang et al., 2024). Although a bit different from these approaches, in Neff et al.’s (2024) study, computational methods were also applied to naturalistic audio recordings, where machine-learning based speaker identification algorithms were used to estimate behavioral indicators such as the length of participants’ own speech, providing a proxy objective measure of everyday communicative activity.

Comprehension and semantic-integration measures were comparatively rare. Only two studies examined semantic processing, reflecting the still-limited evidence on how loneliness relates to language comprehension beyond basic lexical production tasks. Zhou et al. (2022) investigated semantic processing through a lexical decision task targeting lexical ambiguity resolution, examining how perceived loneliness influences the ability to select the appropriate meaning of Chinese characters with multiple sublexical meanings during reading. Li et al. (2025), in turn, they explored the neural dynamics of semantic processing using ERPs, focusing specifically on the N400 component, a well-established electrophysiological marker of semantic access and integration (Kutas and Federmeier, 2011). In their study, participants completed a semantic category verification task, judging whether a target word belonged to a given category (e.g., “sparrow” as a member of “bird”), while electrophysiological activity was recorded.

### 3.5 Main findings regarding language and L/SI

Findings were heterogeneous across constructs, language measures, study designs, and covariate-adjustment strategies. Several studies reported that loneliness predicted poorer verbal fluency (e.g., Beller and Wagner, 2020; Yin et al., 2019), and most studies found a negative association between L/SI and verbal fluency performance (e.g., Lara et al., 2019a; Rodriguez et al., 2017), even after controlling for key variables (e.g., sex, age, education, global cognitive functioning, depressive symptoms, and chronic health conditions). For example, Luchetti et al. (2022) observed significant actor effects (i.e., participant’s own performance) of loneliness on fluency in couples controlling for age, education, and household size, and Lampraki et al. (2025) reported the lowest fluency scores in the “isolated and lonely” profile in multilevel models adjusted for age, sex, education, chronic conditions, and hearing loss. However, in some cases, these associations became non-significant after covariates’ adjustments, as education and depressive symptoms (e.g., DiNapoli et al., 2014). For instance, Shankar et al. (2013) found that the longitudinal association between loneliness and fluency over 4 years became non-significant after adjustment, although social isolation remained a significant predictor. Similarly, Kyaw and Levine (2023) reported that the association between loneliness and semantic fluency did not persist after controlling for age, sex, education, depression, and vascular risk factors, highlighting the importance of multiple contextual variables when interpreting results. Some studies did not find a significative association at all (e.g., Stokes et al., 2023; Okely and Deary, 2019).

Evidence regarding other language domains was limited and inconsistent. Some studies reported no significant associations between L/SI and naming performance after covariates adjustments (Lee et al., 2024; DiNapoli et al., 2014). While some evidence suggests that higher levels of crystallized intelligence, measured through reading tests such as the NART or WTAR, were associated with a lower likelihood of increased loneliness several years later (Okely and Deary, 2019). Participants with lower baseline verbal abilities were nearly twice as likely to report increased loneliness compared with those with higher scores, suggesting that stronger verbal skills may support the maintenance of social engagement (Okely and Deary, 2019). Preliminary evidence suggests that loneliness may be linked to differences in semantic integration and competition resolution, reflected in both behavioral and neural responses (Zhou et al., 2022; Li et al., 2025). Electrophysiological evidence indicates that loneliness is linked to altered neural responses during semantic processing (Li et al., 2025). Specifically, higher loneliness levels were negatively correlated with the reduction in N400 amplitude typically observed during category verification tasks. Participants reporting higher loneliness also exhibited delayed and attenuated N400 responses, suggesting less efficient activation of associative information within long-term semantic memory (Li et al., 2025). Behavioral evidence from lexical decision tasks further indicates that loneliness may be related to difficulties in resolving semantic competition. Individuals with higher loneliness showed greater difficulty establishing semantic relationships between constituent characters in Chinese compound words, resulting in stronger sublexical ambiguity disadvantage effects during word recognition (Zhou et al., 2022).

As for discourse production, evidence suggests that loneliness may influence both the quantity and quality of speech. Several studies reported that higher loneliness levels were associated with increased off-topic verbosity (OTV) in older adults, indicating a tendency toward more tangential or elaborative speech during conversational tasks (Arbuckle et al., 2004; Barnett et al., 2023). Interestingly, some findings suggest that elevated OTV may also serve an adaptive communicative function. When everyday functional competence was taken into account, greater verbosity was associated with lower loneliness levels, possibly reflecting attempts to maintain social interaction through conversational engagement (Arbuckle et al., 2004). In contrast, analyses of naturalistic speech indicated that loneliness was associated with reduced overall speech output, suggesting that lonelier older adults may have fewer opportunities to engage in extended communication (Barnett et al., 2023). Evidence from clinical populations further supports an association between loneliness and discourse abilities. Among older adults with moderate dementia, loneliness explained approximately 8% of the variance in narrative language performance, and the social dimension of loneliness predicted poorer performance on the Narrative Language Test in both mild and moderate dementia (Carbone et al., 2022). Conversely, reduced narrative complexity has also been identified as a predictor of loneliness in older adults, with depressive symptoms partially mediating this relationship (Wang et al., 2025).

Finally, computational linguistics and machine learning approaches have begun to identify speech-based markers of social connectedness. Greater use of first-person plural pronouns (e.g., “we”) was negatively associated with loneliness in women, whereas lonely individuals tended to use more first-person singular pronouns (e.g., “I”), indicating more self-focused language use (Badal et al., 2021b; Wang et al., 2024). Analyses of sentiment and emotional expression further showed that responses from lonely individuals often contained stronger expressions of sadness and negative affect and were typically longer when participants were asked direct questions about loneliness-related experiences (Badal et al., 2021a). Machine learning analyses also indicated that negative sentiment and reduced positivity in speech were associated with lower levels of perceived emotional and instrumental support (Badal et al., 2021b). In addition to lexical and semantic markers, loneliness was associated with several acoustic speech characteristics, including longer pauses, reduced pitch variability, lower formant frequencies, and increased use of fillers or hesitation markers (Yamada et al., 2021). Linguistic features such as sentence complexity and response length also emerged as important predictors in machine learning models trained to detect social disconnection (Badal et al., 2021b; Wang et al., 2024). Evidence from naturalistic speech recordings further suggests that social context and cognitive abilities may influence everyday language production. For example, living without a partner predicted shorter speech duration, whereas higher levels of semantic fluency and cognitive processing speed were strong predictors of how much older adults spoke in daily life (Neff et al., 2024).

## 4 Discussion

This scoping review synthesized the existing literature examining the association between L/SI and language functioning in older adults and mapped the language domains and measures assessed in the studies. Although only 34 studies met the inclusion criteria, three broad patterns already emerged: a) the evidence base is heavily dominated by verbal fluency tasks; b) both L/SI and language are operationalized with considerable heterogeneity; and c) a newer line of work using discourse and naturalistic speech measures appears promising but remains methodologically immature. Many included studies were primarily designed to investigate cognition more broadly rather than language specifically, which helps explain why language was often represented by brief proxy measures embedded within wider cognitive batteries.

Overall, the studies reviewed reveal substantial variability in the linguistic measures used. While a wide range of language domains were represented, including verbal fluency, lexical retrieval, discourse production, semantic processing, and computational linguistic markers, the evidence was strongly dominated by verbal fluency tasks, which were often embedded within broader cognitive batteries rather than designed as dedicated language assessments. This likely reflects several methodological advantages, such as their brief and easy applicability and minimal necessity of materials. It is important to note that many studies describe the use of verbal fluency tasks as a way to assess executive functioning (EF), and not language per se. Literature often describes verbal fluency tasks (phonemic and semantic) as hybrid and multidimensional measures, reflecting the interaction of both language and executive processes (Shao et al., 2014). Verbal fluency tasks require several cognitive mechanisms simultaneously, including lexical retrieval, semantic memory access, and executive processes (e.g., strategic search and response selection, monitoring, and inhibition of repetitions and inappropriate responses; Shao et al., 2014; Taler and Phillips, 2008; Yin et al., 2019). Due to these demands on EF, some authors explicitly use these tasks as an executive function test (Shao et al., 2014; Whiteside et al., 2016). Conversely, other works emphasize the central role of language processes in these tasks. For example, Whiteside et al. (2016) found that both phonemic and semantic fluency loaded primarily on a language factor rather than an executive function factor. For the purpose of this review, verbal fluency tasks were therefore classified as language measures, even when individual studies used them as EF measures. This decision should be understood as a pragmatic classification reflecting the linguistic demands of these tasks, rather than as an assumption that verbal fluency is a process-pure index of language.

In contrast, more complex linguistic domains such as discourse organization, semantic integration, and naturalistic speech analysis were comparatively underrepresented. Notably, recent studies have begun to incorporate computational approaches and natural language analyses, allowing researchers to capture subtle linguistic and communicative patterns that extend beyond traditional performance-based tests. These findings highlight a field that remains largely anchored in brief neuropsychological indicators and laboratory-based cognitive tasks, which may not fully capture the complexity of language use in everyday social interactions. In contrast, emerging methodologies offer promising avenues for examining language functioning in a more ecologically valid context.

Taken together, the available evidence is more suggestive than definitive. Verbal fluency is the most frequently studied domain and often shows poorer performance in individuals with higher loneliness or social isolation, but these associations are not uniformly robust after adjustment for education, depressive symptoms, health status, and global cognition (e.g., DiNapoli et al., 2014; Gilmour, 2011; O’Luanaigh et al., 2012). Evidence for lexical retrieval and naming is sparse and inconsistent, whereas discourse- and speech-derived measures may be more sensitive to the lived social consequences of disconnection. The small number of studies on semantic processing and naturalistic language markers is encouraging, but still insufficient to support strong conclusions. These results indicate not simply that language has been understudied, but that it has often been measured too narrowly. Despite the central role of language in social interaction, relatively few studies examined higher-level linguistic processes such as discourse organization, pragmatic appropriateness, semantic integration, or everyday communicative behavior. Future work would benefit from richer language phenotyping, including narrative and conversational tasks, pragmatic measures, and repeated naturalistic speech sampling. Such approaches may offer a more ecologically valid account of how social disconnection is reflected in communication.

Despite the small number of studies, one promising avenue has been the identification of linguistic features derived from natural speech such as pauses, pitch, response length, sentiment, using NLP and machine learning (ML) approaches (e.g., Badal et al., 2021a, 2021b; Yamada et al., 2021). Overall, these studies suggest that naturalistic language analysis is a promising exploratory avenue for studying social disconnection in older adults and may support the identification of individuals with higher levels of L/SI, with reported classification accuracy for high vs. low L/SI ranging from 73 to 96% (Badal et al., 2021a, 2021b; Yamada et al., 2021). These findings should nevertheless be interpreted cautiously, given the small samples, task specificity, and still limited external validation. Several arguments support the integration of NLP and ML approaches into research on L/SI in aging. Linguistic features are known to be modulated by emotional states, and may therefore provide relevant cues about individuals’ affective experience (Hashem et al., 2023). In addition, linguistic markers have been explored as putative indicators of psychiatric symptoms, including depression and suicidality, with potential applications in the diagnosis, monitoring and treatment of mental health conditions (Low et al., 2020). A similar trend has been observed in aging research, driven by the possibility of detecting early signs of cognitive decline and dementia through speech and language features (Gagliardi, 2024).

Naturalistic speecPh analysis may also address some limitations of conventional self-report measures of L/SI. Some self-report instruments avoid overt references to loneliness or social isolation in order to reduce stigma-related response bias and underreporting. Language-based approaches may be consistent with this concern, while also going further by reducing reliance on introspective report and memory-based self-assessment. They may therefore help mitigate biases associated with subjective reporting, including social desirability, recall bias, and gender-related response patterns, while remaining flexible enough to capture heterogeneity in L/SI experiences across subgroups and generations. Despite this encouraging evidence, further studies are needed in culturally diverse community samples, and testing other L/SI assessment approaches specifically designed or adapted for older adult populations.

Beyond the diversity of language measures, there was marked heterogeneity in study populations, follow-up intervals, cultural contexts, L/SI measures, and analytic strategies. This heterogeneity, together with the uneven handling of covariates across studies, helps explain why a meta-analysis was not appropriate and why direct comparison across findings remains difficult. The clinical status of participants represents an additional source of heterogeneity. Included studies ranged from community-dwelling older adults to individuals with mild cognitive impairment or dementia, and recent evidence suggests that associations between loneliness and cognitive or functional outcomes may differ across minor and major neurocognitive disorder populations (Moretti et al., 2024). This reinforces the need for caution when comparing findings across cognitively healthy, mildly impaired, and dementia samples.

Regarding L/SI measures, substantial heterogeneity was also observed across studies. Only a small subset assessed both constructs simultaneously, limiting our ability to examine their independent and combined effects with language-related outcomes. This is relevant because loneliness and social isolation, although related, are conceptually distinct constructs (Hawkley and Cacioppo, 2010; Holt-Lunstad et al., 2015), supported empirically by works showing small to moderate correlations between them (Coyle and Dugan, 2012; Holt-Lunstad et al., 2015; McHugh et al., 2017), and partly distinct associations with cognitive outcomes (Cardona and Andrés, 2023; Kang et al., 2024). For this reason, profile-based approaches may be particularly informative. Menec et al. (2020), for example, emphasized the importance of distinguishing between individuals who are neither isolated nor lonely, lonely but not isolated, isolated but not lonely, and both isolated and lonely. This approach is also illustrated by Lampraki et al. (2025), who found that individuals in more socially disadvantaged profiles performed consistently worse across cognitive domains than those in the non-isolated and not lonely profile. Such profiles capture different combinations of subjective and objective social disconnection and may therefore provide a nuanced understanding of risk than either construct than either construct considered in isolation.

How L/SI are measured is also central to interpretability. For loneliness, although the UCLA Loneliness Scale (Russell, 1996; Russell et al., 1978, 1980), and the De Jong Gierveld scales (De Jong-Gierveld and Kamphuis, 1985; De Jong Gierveld and Van Tilburg, 2006; De Jong Gierveld and Van Tilburg, 2010) were commonly used, the literature also includes single-item questions and other brief indicators such as “Do you feel lonely?” or “Do you feel very lonely?.” Mixing direct single-item questions with multidimensional scales likely contributes to inconsistent findings, because these instruments differ in scope, sensitivity, and vulnerability to underreporting (Yanguas et al., 2018). Loneliness is a sensitive and stigmatized concept, and direct questions often lead to underreporting (Boss et al., 2015). Furthermore, less comprehensive measures may fail to capture the complexity of loneliness experiences and may be less sensitive to detecting associations between loneliness and subsequent cognitive decline (Okely and Deary, 2019). Comparable measurement variability was observed for social isolation. Some studies used a social isolation index or composite indicators combining multiple dimensions of social contact and participation (e.g., Shankar et al., 2013), whereas others used validated multi-item instruments, such as the Lubben Social Network Scale (e.g., Rodriguez et al., 2017). Other studies focused on broader social connection constructs, such as social participation (e.g., Gilmour, 2011), or relied on single indicators, such as marital status (Estrella et al., 2024). These differences matter because social isolation, social network size, social participation, marital status, and broader social connection are related but not interchangeable constructs. Future studies should favor validated multi-item instruments and be cautious when comparing results across different measurement traditions. Future research should also more clearly distinguish loneliness, social isolation, and broader social connection constructs and measures (Gardam et al., 2023; Perissinotto et al., 2019); report covariate choices transparently; and prioritize longitudinal designs capable of disentangling directionality and reciprocal effects.

Another important aspect is the cultural and linguistic context of the studies. Most were conducted in Western, high-income countries, particularly Europe and North America. Cultural and linguistic differences may influence both language use and experiences of L/SI, as illustrated by Beller and Wagner (2020) regarding cultural individualism. More cross-cultural research is therefore needed, and future studies should examine how associations between L/SI and language may vary across languages, sociolinguistic contexts, and individuals’ linguistic backgrounds, including native-language characteristics and monolingual versus multilingual experience. Speech- derived measures, combined with computational and NLP approaches, may be particularly useful in this regard because they can be adapted to culturally and linguistically diverse communities assuring that models are appropriately validated within each context.

The exclusion of primarily auditory speech-perception measures, such as speech-in-noise tasks, should also be interpreted as a boundary of the current review rather than as a claim that such tasks are unrelated to language. Speech comprehension in adverse listening conditions is an important auditory-cognitive-linguistic domain and may be highly relevant to social participation in older adulthood. However, including this literature would have required a broader review integrating hearing status, auditory perception, listening effort, working memory, and speech comprehension, as well as the links between hearing loss, social participation, and cognitive decline, topics that have been addressed in prior reviews and theoretical frameworks (e.g., Maharani et al., 2019; Shukla et al., 2020).

In conclusion, this review maps a literature that is still fragmented, heavily centered on verbal fluency, and only beginning to move toward richer discourse and naturalistic speech approaches. Current evidence suggests that L/SI may be associated with aspects of language functioning in older adults, but the field is not yet mature enough to support simple or uniform conclusions. Expanding research beyond brief neuropsychological indicators toward more ecologically valid and naturalistic language assessments may provide a deeper understanding of how social experiences shape communication in aging. Progress will depend on clearer construct separation and measurement (Gardam et al., 2023; Perissinotto et al., 2019), more precise language measurement, and stronger longitudinal designs. Ultimately, understanding how language reflects and shapes social connectedness in older adulthood may open new avenues for identifying individuals at risk of social disconnection and for developing communication-based approaches to promote healthy aging.

## Data Availability

The original contributions presented in the study are included in the article/Supplementary material, further inquiries can be directed to the corresponding author.
