# Supplementary material for: From verbal fluency to naturalistic speech: a scoping review of language correlates of loneliness and social isolation in older adults
Source: Front Aging Neurosci. 2026 Jul 31;18:1831374. doi: 10.3389/fnagi.2026.1831374 (PMC13473163; doi:10.3389/fnagi.2026.1831374)
Supplement: Supplementary file 1 [file Data_Sheet_1.DOCX]

**Supplementary Materials**

Table S1

*Databases Search Expressions and Exclusion Filters*

| **Search expressions** | **Document-type exclusion filters** |
| --- | --- |
| Web of Science:  ((TS=("aging" OR "ageing" OR elder* OR older* )) AND TS=("speech" OR "language" OR linguistic* OR verbal*)) AND TS=("loneliness" OR "lonely" OR "social isolation") | Review Article; Meeting; Abstract; Letter; Editorial Material; Correction; Retracted Publication |
| PubMed:  (("aging"[Title/Abstract] OR "ageing"[Title/Abstract] OR elder*[Title/Abstract] OR older*[Title/Abstract]) AND ("speech"[Title/Abstract] OR "language"[Title/Abstract] OR linguistic*[Title/Abstract] OR verbal*[Title/Abstract])) AND ("loneliness"[Title/Abstract] OR "lonely"[Title/Abstract] OR "social isolation"[Title/Abstract]) | Address; Autobiography; Bibliography; Biography; Books and Documents; Clinical Conference; Clinical Trial Protocol; Clinical Trial, Phase I; Clinical Trial, Phase II; Clinical Trial, Phase III; Clinical Trial, Phase IV; Clinical Trial, Veterinary; Comment; Congress; Consensus Development Conference; Consensus Development Conference, NIH; Dataset; Dictionary; Directory; Duplicate Publication; Editorial; Electronic Supplementary Materials; English Abstract; Equivalence Trial; Expression of Concern; Festschrift; Interactive Tutorial; Interview; Lecture; Legal Case; Legislation; Letter; Meta-Analysis; Network Meta-Analysis; News; Newspaper Article; Observational Study, Veterinary; Preprint; Published Erratum; Randomized Controlled Trial, Veterinary; Retracted Publication; Retraction of Publication; Review; Scientific Integrity Review; Scoping Review; Systematic Review; Technical Report; Video-Audio Media; Webcast. |
| Scopus:  (TITLE-ABS-KEY("aging" OR "ageing" OR elder* OR older*) AND TITLE-ABS-KEY("speech" OR "language" OR linguistic* OR verbal*) AND TITLE-ABS-KEY("loneliness" OR "lonely" OR "social isolation")) | Review; Conference paper; Book chapter; Editorial; Note; Conference review; Letter; Short survey; Book; Data paper; Erratum. |

Table S2.

*Studies Assessing Only Loneliness*

| **Loneliness measure(s)** | | | **Studies** |
| --- | --- | --- | --- |
| UCLA Loneliness Scale (only) | | 20-item (V3) | Li et al. (2025), Yamada et al. (2021), Zhou et al. (2022) |
|  |  | 11-item | Stokes et al. (2023) |
|  |  | 3-item | Beller & Wagner (2020), Huang et al. (2025), Luchetti et al. (2022), Wang et al. (2025), Yin et al. (2019) |
| 20-item UCLA Loneliness Scale + semi-structured interview questions | | | Badal, Graham et al. (2021),  Wang et al. (2024) |
| 20-item UCLA Loneliness Scale + single item (Center for Epidemiologic Studies-Depression Scale - CES-D). | | | Lee et al. (2024) |
| Three-Item Loneliness Scale (TILS) | | | Barnett et al. (2023) |
| 6-item Social and Emotional Loneliness Scale | | | Carbone et al. (2022) |
| 6-item de Jong Gierveld Loneliness Scale | | | Domenicucci et al. (2025) |
| Single item | “During the past week, how often have ‘I felt lonely’” (from CES-D) | | Kyaw & Levine (2023) |

Table S3.

*Studies Assessing Both Loneliness and Social Isolation (L/SI)*

| **L/SI Measures** | **Studies** | **L Details** | **SI Details** |
| --- | --- | --- | --- |
| 3-item UCLA Loneliness Scale + Social Isolation Index | Shankar et al. (2013)  Lara, Caballero, et al. (2019) | This scale assesses loneliness by asking participants about the frequency of feeling a lack of companionship, feeling left out, and feeling isolated from others. | 5-item Social Isolation Index (marital status, monthly contact with children, family, friends, and participation in social organizations). |
|  | Bethell et al. (2024) |  | Adapted version of the 5-item Social Isolation Index with different thresholds (e.g., contact within the past 6 months) and an additional indicator related to low participation in social activities among retirees. |
| 3-item UCLA Loneliness Scale + Social Connectedness Scale | Lampraki et al. (2025) |  | The Social Connectedness Scale is a composite summary (0–4) provided by the SHARE study, incorporating network size, proximity (within 25 km), frequency of contact (at least weekly), support (close emotional ties), and diversity (types of relationships). |
| 6-item de Jong-Gierveld Loneliness Scale + 18-item Lubben Social Network Scale (LSNS-18) | Schnittger et al. (2012) | This scale distinguishes between two types of loneliness: social loneliness, which refers to the perceived absence of a broader social network or sense of community, and emotional loneliness, which refers to the absence of a close, intimate, or attachment relationship | LSNS-18 examines perceived family, friendship and neighborhood social support and, from this, gauges levels of social isolation. |
